# Ocean Acidification Affects Redox-Balance and Ion-Homeostasis in the Life-Cycle Stages of *Emiliania huxleyi*

**DOI:** 10.1371/journal.pone.0052212

**Published:** 2012-12-26

**Authors:** Sebastian D. Rokitta, Uwe John, Björn Rost

**Affiliations:** Alfred Wegener Institute for Polar and Marine Research, Bremerhaven, Germany; University of Gothenburg, Sweden

## Abstract

Ocean Acidification (OA) has been shown to affect photosynthesis and calcification in the coccolithophore *Emiliania huxleyi*, a cosmopolitan calcifier that significantly contributes to the regulation of the biological carbon pumps. Its non-calcifying, haploid life-cycle stage was found to be relatively unaffected by OA with respect to biomass production. Deeper insights into physiological key processes and their dependence on environmental factors are lacking, but are required to understand and possibly estimate the dynamics of carbon cycling in present and future oceans. Therefore, calcifying diploid and non-calcifying haploid cells were acclimated to present and future CO_2_ partial pressures (*p*CO_2_; 38.5 Pa vs. 101.3 Pa CO_2_) under low and high light (50 vs. 300 µmol photons m^−2^ s^−1^). Comparative microarray-based transcriptome profiling was used to screen for the underlying cellular processes and allowed to follow up interpretations derived from physiological data. In the diplont, the observed increases in biomass production under OA are likely caused by stimulated production of glycoconjugates and lipids. The observed lowered calcification under OA can be attributed to impaired signal-transduction and ion-transport. The haplont utilizes distinct genes and metabolic pathways, reflecting the stage-specific usage of certain portions of the genome. With respect to functionality and energy-dependence, however, the transcriptomic OA-responses resemble those of the diplont. In both life-cycle stages, OA affects the cellular redox-state as a master regulator and thereby causes a metabolic shift from oxidative towards reductive pathways, which involves a reconstellation of carbon flux networks within and across compartments. Whereas signal transduction and ion-homeostasis appear equally OA-sensitive under both light intensities, the effects on carbon metabolism and light physiology are clearly modulated by light availability. These interactive effects can be attributed to the influence of OA and light on the redox equilibria of NAD and NADP, which function as major sensors for energization and stress. This generic mode of action of OA may therefore provoke similar cell-physiological responses in other protists.

## Introduction

The dissolution of carbon dioxide (CO_2_) in the oceans and the resulting formation of carbonic acid are causing a chemical shift towards higher [CO_2_] and acidity ([H^+^]), a phenomenon known as ocean acidification (OA) [1]. OA has been demonstrated to affect metabolic processes and especially calcification in numerous marine organisms [2], including coccolithophores. Like other phytoplankton, these bloom-forming unicellular algae sustain vertical gradients of dissolved inorganic carbon (DIC) by the formation of particulate organic carbon (POC, i.e., biomass) and its subsequent depth-export (organic carbon pump) [3]. Additionally, calcification and the export of particulate inorganic carbon (PIC, i.e., CaCO_3_) maintain vertical gradients of alkalinity in the oceans (inorganic carbon pump) [3]. The CaCO_3_ mineral furthermore ballasts organic matter aggregates and enhances their export [4], [5], thereby influencing the oceanś capacity to sequester carbon [6]–[8]. Insights into the cell-biology of biomass production and calcification and their dependence on environmental factors are required to understand and possibly estimate the dynamics of carbon cycling in present and future oceans.

For more than a decade, *Emiliania huxleyi*, the most abundant coccolithophore, has been intensively investigated towards its susceptibility to OA [9], [10] and the functioning of its carbon concentrating mechanism (CCM) [11]–[13]. In experiments, cells were usually acclimated to a range of different OA-scenarios with controlled carbonate chemistry before assessing elemental composition and physiological parameters. Although partly contradictive results were obtained between species and strains [14], most *E. huxleyi* datasets showed an overarching pattern of increased or unaltered production of POC accompanied by a reduced or unaltered PIC production, typically reflected by a decreased PIC:POC ratio [9], [15], [16]. Bach et al. [17] observed that PIC and POC production possess different [CO_2_] optima, the lower boundary being defined by limitation of inorganic carbon (C_i_) whereas the upper boundary is restricted by detrimental [H^+^]. This adds support to the idea that both processes operate largely independent and consequently are in competition for energy and C_i_ in the cell.

In recent studies, so-called matrix approaches were used, in which the effects of OA were investigated in combination with other, independently varied parameters like light, temperature, or nutrients [18]–[21]. These studies revealed that the effects of OA on different physiological parameters vary significantly, depending on the constellation of framing environmental parameters, which directly or indirectly relate to cellular energy state (Fig. 3 in [16]). Also, the often neglected haploid life-cycle stage has attracted interest as it appears to play an intriguing role in this specieś ecology. For instance, haplonts are resistant to attacks of stage-specific viruses that diminish blooms of diploid individuals, so that meiosis is believed to be an ecological escape strategy [22]. Besides this, the haplo-diplontic life-cycle provides a unique model system in which calcification can be studied in two functionally different stages that share the same genetic material. To characterize the energy dependence of OA-effects in the haploid and diploid life-cycle stages of *E. huxleyi* (RCC 1216 and RCC 1217, respectively), cells were acclimated to an experimental matrix of present-day vs. elevated [CO_2_] (38.5 Pa vs. 101.3 Pa CO_2_, corresponding to ∼380 vs. ∼1000 µatm and yielding [CO_2_] of ∼14 vs. ∼39 µmol kg^−1^) under low and high light intensities (50 vs. 300 µmol photons m^−2^ s^−1^) [16]. This study found that the diploid stage shunted resources from PIC towards POC production under OA, yet keeping the production of total particulate carbon constant. In the haploid stage, major physiological rearrangements, like changes in the photosynthetic apparatus, were evident but resulting elemental composition and production rates were more or less unaffected by OA. Both life-cycle stages appear to pursue distinct strategies to deal with altered carbonate chemistry. As a general pattern, OA-responses were strongly modulated by energy availability and typically most pronounced under low light [16].

In the present study, microarray-based gene expression data are presented that originate from RNA samples from that very experiment. This comparative approach not only enables the investigation of OA-responses and their energy-dependent modulation on a transcriptomic level, but also the advancement of functional interpretations derived from existing physiological data [16]: As OA affected the allocation of carbon and enhanced cellular energy efficiency, the presented analyses focused on genes related to carbon metabolism and light physiology. In addition, since increased acidity under OA affects membrane potentials [23], [24], genes related to signal transduction and ion fluxes were examined. Lastly, the responses of the life-cycle stages were compared to elaborate on commonalities and peculiarities of their OA-responses.

## Results and Discussion

The responses to OA and their modulation by light intensity were examined in the calcifying and non-calcifying life-cycle stages of *Emiliania huxleyi*. Transcriptomic data was mined for indicative cellular functions. Findings were discussed in the context of previously obtained physiological data to resolve the effects of OA on the sub-cellular interplay between processes that generate energy (photosynthetic light reactions) and those that compete for it (biomass buildup and calcification). In the following, mentioned up- or down-regulation (in response to OA and/or high light) is always related to the respective opposite treatment (i.e., present-day *p*CO_2_ and/or low light). For scope and clarity, more emphasis was put on the effects of OA rather than on the effects of high light intensity.

### The Diploid Stage

**Figure 1:**
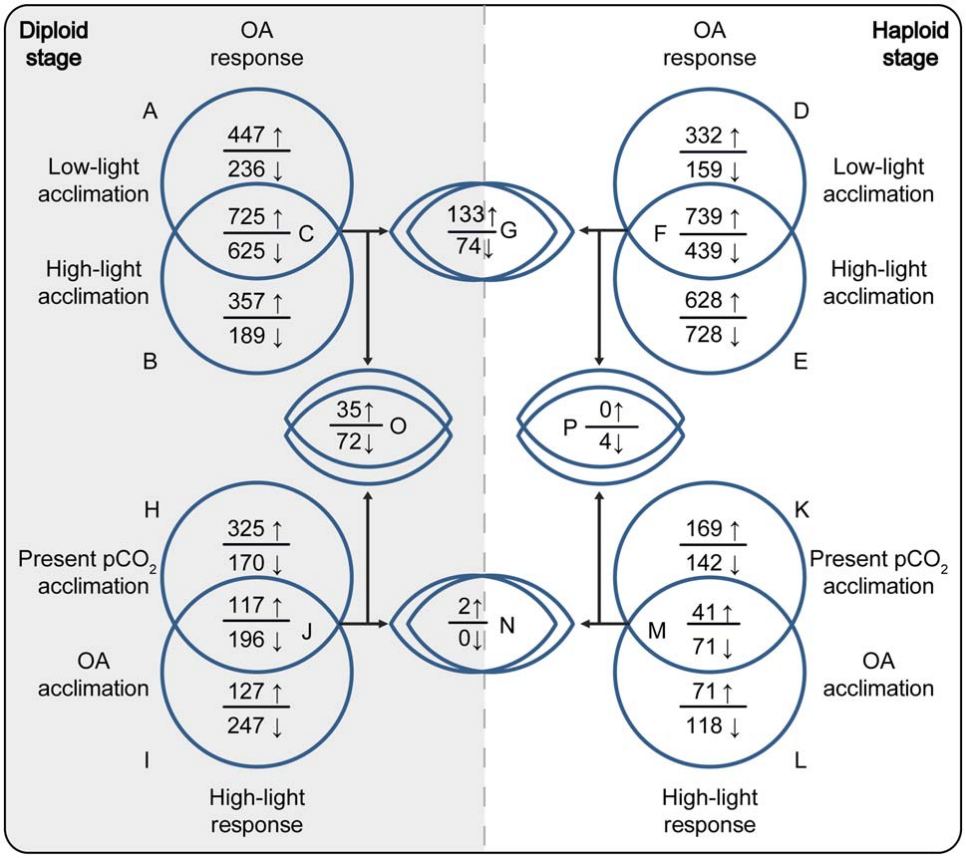
Sets and subsets of differential gene expression in *Emiliania huxleyi* in response to Ocean Acidification and high light intensity. Responses to Ocean Acidification (upper part) and high light intensity (lower part) are shown for the diploid (left part, shaded) and the haploid (right part) life-cycle stage. Numbers represent significantly regulated genes; arrows indicate up- or down-regulation (↑ or ↓).

**Figure 2:**
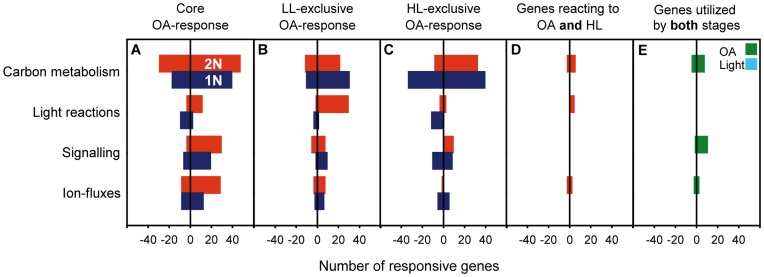
Numbers of responsive genes in the categories ‘carbon metabolism’, ‘light reactions’, ‘signaling’ and ‘ion-fluxes’. Sign indicates up- or down-regulation (+ or −); LL and HL denote low-light and high-light specificity of responses.

In low-light acclimated cultures, OA significantly altered the expression of 2033 genes (1172 up-regulated (↑) and 861 down-regulated (↓), respectively; Fig. 1A). In the high-light acclimated cultures, 1896 genes were significantly regulated in response to OA (1082↑ and 814↓; Fig. 1B). The intersection of sets A and B yielded the diplont-specific ‘core OA-response’ with 1350 significantly CO_2_-regulated genes (725↑ and 625↓; Fig. 1C). The visual inspection of this subset revealed 158 genes (115↑ and 43↓) that could be assigned to the predefined categories ‘carbon metabolism’, ‘light physiology’, ‘signaling’ and ‘ion fluxes’ (Fig. 2A; Table S1C).

#### General OA-responses

Expression of genes of the primary carbon metabolism was prominently stimulated under OA (Fig. 2A; Table S1C), including trehalose-6-phosphate synthase/phosphatase (GJ27270) and genes relevant in glycolysis (GL), e.g., glucose-6-phosphate isomerase (GJ06821), phosphoglyceratkinase (GJ28540) and phosphoglycerate mutase (GJ25367). Also, expression of genes of the pentose phosphate pathway (PPP) was stimulated by OA, e.g., glucose-6-phosphate dehydrogenase (GJ12738), 6-phosphogluconolactonase (GJ20503) or ribulose-5-phosphate epimerase (GJ03996). The induction of trehalosephosphate synthase/phosphatase points towards a decreased activity of glycolysis (GL), as this enzyme is considered an important pacemaker of cytoplasmic carbohydrate breakdown [25]. Furthermore, the up-regulation of several other regulatory enzymes involved in GL ([26], [27]) and the PPP ([28], [29]) under OA suggests altered fluxes of carbon through the metabolism: GL and PPP are the main pathways competing for cytoplasmic glucose-6-phosphate. GL and subsequent oxidative reactions of the mitochondrial tricarboxylic acid cycle generate NADH mostly destined for respiration. The PPP, in contrast, can operate within several flux modes to satisfy different demands, especially for NADPH required in anabolic, reductive processes like storage-compound synthesis [30]. Under OA, cells obviously increase the relative activity of the PPP and thereby redirect the metabolic carbon fluxes.

Numerous genes involved in the turnover of polysaccharides were up-regulated in response to OA, e.g., callose synthase (GJ16141) and glucan beta-1-3 glucosidase (GJ14154). Genes related to intra- and extracellular glycosylation, e.g., N-acetylglucosamine transferase (GJ14803) or N-acetylneuraminate transferase (GJ03189) were found to be differentially expressed. Lipid-synthesizing machinery was induced under OA, e.g., 3-oxoacyl-(ACP) synthases (GJ00191, GJ09435). Production of carbohydrates, especially chrysolaminaran-like β-1,3-glucans (Table S1C), provides a means of storing excess energy and carbon in situations of high photosynthate production, e.g., high light [31] and also under OA [32]. Especially the induction of lipid synthesis together with the observed up-regulation of the PPP advocates increased accumulation of storage compounds under OA, which is in line with the observed increased POC quotas [16].

**Figure 3:**
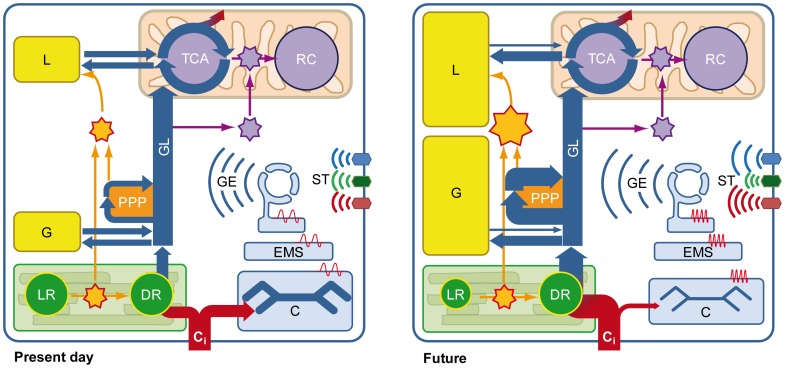
Proposed metabolic constellations of diploid *Emiliania huxleyi.* Under **present-day conditions** (∼38.5 Pa CO_2_), calcification (C) in the coccolith vesicle, and photosynthetic dark reaction (DR) occurring in the chloroplast compete for acquired inorganic carbon (C_i_, red). DR fixes C_i_ into organic carbon (blue) that is channeled through glycolysis (GL) towards mitochondrial oxidation in the tricarboxylic acid cycle (TCA). NADH (purple star) produced in GL and TCA is used to drive mitochondrial ATP generation at the respiratory chain (RC). NADPH (orange star) created in the pentose phosphate pathway (PPP) and photosynthetic light reactions (LR) is used to produce reduced storage compounds like glucans (G) and lipids (L). Under **future** **conditions** (∼101.3 Pa CO_2_), gene expression (GE) is altered and calcification is reduced, probably in consequence of altered plasmalemmal signal transduction (ST) and action-potential signaling at the endomembrane system (EMS). Due to diminished calcification under Ocean Acidification, DR produces more organic carbon. In addition to the intense buildup of glucans, the stimulated activity of the PPP under Ocean Acidification provides more NADPH and promotes lipid synthesis. Enhanced cytosolic levels of NADPH, in turn, result in a down-regulation of NADPH production in the LR.

Regarding light reactions, a prominent up-regulation of fucoxanthin-chlorophyll a/c binding proteins (FCPs), i.e., light harvesting antennae (GJ16045, GJ03834, GJ04497, GJ06058) and genes related to carotenoid biosynthesis, e.g., phytoene desaturases (GJ07905, GJ10400) was observed (Fig. 2A, Table S1C). This indicates higher turnover of light-harvesting antennae and intensified xanthophyll cycling. The latter serves to dissipate excess light energy and reductive pressure [33], which is in line with the observation that *E. huxleyi* downscales light harvesting under OA [16]; [34]. The absence of this regulation pattern from the light-response (i.e., Fig. 1J) indicates a clear causal relationship between the observed phenomena and OA. In line with this, intensified energy dissipation under OA was also observed in natural, diatom-dominated communities [35]. Enhanced dissipation of light energy under OA seems counterintuitive because an increased production of POC [16] as well as increased expression of lipid synthesizing machinery (Table S1C) was observed, processes that should counteract the accumulation of NADPH. The down-regulation of light harvesting can, however, be explained by a decreased cellular demand for NADPH due to up-regulation of the also NADPH-generating PPP. These results show that OA causes a reconstellation of metabolic flux networks (especially GL and PPP) that consequently affects the redox-equilibria of NAD and NADP. These equilibria are sensors of various environmental parameters, control organelle activity [28], [36], and these compounds can be derivatized to function as second messengers in a number of viable cellular processes [37]. Apparently, OA affects this central sensory system and, possibly by increasing the concentrations of carbon precursors and NADPH, causes cells to shunt relatively more carbon into reduced storage compounds (Fig. 3). This mechanism explains the widely observed increase in POC production under OA [e.g., 9, 16, 38].

Genes related to cellular signaling, e.g., diverse phosphatidylinositolphosphate kinases (PIPKs; GJ04870, GJ03242, GJ22616, GJ02707), sphingosine-1-kinases (GJ24982, GJ10937) and associated downstream signaling kinases, so-called ‘CBL-interacting proteins’ (GJ14428, GJ00645, GJ01417, GJ01704) were prominently up-regulated (Fig. 2A, Table S1C). Apparently, OA alters signal transduction, affecting diverse actors in plasmalemma-situated signaling cascades that involve second messengers like inositolphosphates and sphingosine. These primarily control Ca^2+^ effluxes from the endoplasmic reticulum [39]–[41] and have been implicated in the response to ionic/osmotic stress [42] and apoptosis [43]. In line with this, cells were found to regulate the expression of ion transporters (Fig. 2A, Table S1C): OA induced up-regulation of diverse cellular ion exchangers, e.g., a Ca^2+^/H^+^ antiporter (GJ01185), a Ca^2+^-transporting ATPase (GJ01493), a Na^+^/H^+^ exchanger (GJ10603) as well as a K^+^-dependent Ca^2+^/Na^+^ exchanger (GJ00579). The dataset also showed that distinct HCO_3_^−^ transporters were regulated in response to OA, two being up-regulated (GJ07173 and GJ00654), while one HCO_3_^−^ transporter with an interspersed Ca^2+^-binding EF-hand motif (GJ14380) was down-regulated. The Ca^2+^/H^+^ exchanger was examined earlier (CAX4; [44]) and was attributed to general ion-homeostasis rather than explicit coccolith production. The HCO_3_^−^transporters regulated under OA were earlier described as OA-insensitive [45]. In that study, however, the unaffected expression coincided with unaffected production rates of PIC and POC. In the present experiment, the expression of HCO_3_^–^transporters as well as PIC:POC ratios were clearly affected by OA [16], which is in line with their hypothetical involvement in calcification. As these HCO_3_^–^transporters are situated in the plasma membrane as well as the plastid, it is yet not possible to relate all of them exclusively to the process of calcification. The regulation of energy-dependent ion-transporters clearly shows that altered ion fluxes are required under OA to establish sufficient membrane gradients and currents. In addition, a number of channels were regulated under OA that do work passively and react to transient action potentials. This suggests that the ultimately resulting signals are themselves modulated spatially, temporally as well as in amplitude, probably being a major reason for the impaired calcification commonly reported for diploid *E. huxleyi* under OA [9], [15], [18]. The prominent regulation patterns related to cellular signaling and ion-homeostasis did not occur in response to light intensity (i.e., Fig. 1H, 1I), proving that these effects are exclusively caused by OA. All in all, OA apparently not only affects the signaling networks that facilitate environmental sensing and control cellular ion-gradients but it also affects ion-transport itself (Fig. 3). Fluctuating or offset [Ca^2+^] or [H^+^] as well as possibly extensive glycosylation in the coccolith vesicle seem to be major reasons for the impaired PIC production typically observed in coccolithophores under OA [24], [44].

#### Energy-modulation of OA-responses

To examine particularly the energy-modulation of OA-effects in the diploid stage, the subset C (i.e., the core response) was subtracted from sets A and B (termed ‘A\C’, ‘B\C’). This subtraction yields only those OA-responses that occur exclusively under either low or high light levels (Fig. 1A and 1C, 1B and 1C; Table S1A and S1C, S1B and S1C). These genes, being comprised only in either subset B or A (i.e., *low-light-specific* and *high-light-specific* OA-responses; Fig. 1), likely represent those cellular functions that underlie the observed light-modulation of OA-responses in the diploid stage [16].

The low-light specific OA-response showed significant regulation of 683 genes (447↑ and 236↓; Fig. 1A and 1C; Table S1A and S1C). Visual inspection of this group revealed 84 genes (64↑ and 20↓) that could be assigned to the categories of interest (Fig. 2B): Concerning carbon metabolism, there was up-regulation of cytoplasmic fructose-2,6-bisphosphatase (GJ04837) and plastidic fructose-1,6-bisphosphatase (GJ09341) especially under low-light conditions. Malate dehydrogenase (GJ02937) and cytoplasmic pyrophosphate-dependent fructose-1,6-bisphosphatase (GJ01131) were found down-regulated in response to OA under low light. These enzymes play catalytic and regulatory roles in GL and PPP and also control cellular levels of trioses, hexoses and inorganic phosphate [46], [47]. The observed down-regulation of NAD-dependent malate dehydrogenase in response to OA, especially under low light, indicates reduced shuttling of reduction equivalents between cytoplasm and the mitochondrial matrix. This may be attributed to elevated [CO_2_] affecting respiratory processes [36], [48] and the overall reconstellation of the redox state. Genes related to light reactions, e.g., zeaxanthin epoxidases (GJ05220, GJ09052), violaxanthin de-epoxidase (GJ01361) as well as FCPs (GJ26033, GJ04236, GJ04295, GJ04849, GJ07716, GJ08463) were concertedly up-regulated under OA. This indicates a more intense xanthophyll cycling, i.e., energy-dissipation in response to OA, especially under low-light. No pronounced expression shifts could be recognized for genes related to ‘signaling’ or ‘ion fluxes’, besides one distinct HCO_3_^−^ transporter (GJ15963), which was found up-regulated.

In the high-light specific OA-response, significant regulation of 546 genes (357↑ and 189↓; Fig. 1B and 1C; Table S1B and S1C) was observed. Visual inspection revealed 55 regulated genes (43↑ and 12↓) that could be assigned to the categories of interest (Fig. 2C). Several genes related to carbon metabolism were affected, namely there was up-regulation of a cytoplasmic fructose-2,6-bisphosphatase (GJ10649) and a number of organellar transporters for primary metabolites, e.g., mitochondrial tricarboxylate/dicarboxylate carriers (GJ03542, GJ20481) and putatively plastidic glucose-6-phosphate/phosphate and phosphoenolpyruvate/phosphate antiporters (GJ09586, GJ16251). These transporters not only connect the primary metabolic pathways of the organelles and the cytosol, but also shuttle reduction equivalents [49], [50] and/or Acetyl-CoA, for instance in the context of the mitochondrial citrate-shuttle [51].

These results indicate that, when energy availability is low, cells under OA tightly regulate the allocation of organic carbon and hence organelle activity. Reduced expression of respiration-relevant genes, especially under low light, and the stimulated synthesis of storage compounds caused by OA, can therefore explain the relatively larger stimulation in POC production under these conditions (+84%; [16]). Increased OA-induced xanthophyll-cycling, especially under low light, can be explained by the NADPH surplus originating from increased PPP activity. Again, this surplus is relatively larger under low light, which agrees with the concept of energy-modulated responses (Fig. 3 in [16]). In turn, when energy availability is high, cells enhance fluxes of metabolites and energy between compartments by up-regulating corresponding transporters.

### The Haploid Stage

In the low-light acclimated haploid cultures, OA significantly altered the expression of 1669 genes (1071↑ and 598↓; Fig. 1D; Table S1D). Under high-light, 2534 genes were significantly regulated in response to OA (1367↑ and 1167↓; Fig. 1E, Table S1E). The intersection of sets D and E yielded the haplont-specific core OA-response with 1178 significantly regulated genes (739↑ and 439↓; Fig. 1F; Table S1F). The visual inspection of this subset revealed 112 genes (72↑ and 40↓) that could be assigned to the categories of interest (Fig. 2A).

#### General OA-responses

Concerning carbon metabolism, OA caused up-regulation of trehalose-6-phosphate synthase/phosphatase (GJ20366) as well as glycolytic enzymes, e.g., plastidic phosphoglucomutase (GJ12007), enolase (GJ01695) and lactate dehydrogenases (GJ12287, GJ17732). Genes of the PPP were found up-regulated, e.g., glucose-6-phosphate dehydrogenase (GJ04421) and 6-phosphogluconolactonase (GJ09221). Down-regulation was observed for plastidic fructose-1,6-bisphosphatase (GJ09341), plastidic phosphoribulokinase (GJ19188) and for functionally different forms of cytosolic glyceraldehyde-3-phosphate dehydrogenase (NADPH-producing, non-phosphorylating GJ03103; NADH-producing, phosphorylating GJ25044). These expression patterns indicate a redox-related reconstellation of carbon flux networks similar to the diplont. Down-regulation of plastidic phosphoribulokinase, the enzyme synthesizing ribulose-1,5-bisphosphate (substrate of RubisCO), indicates a throttling of photosynthetic dark reactions under OA. The up-regulation of lactate dehydrogenases shows that OA stimulates also fermentative reactions to regenerate cytosolic NAD^+^. The NADPH-producing glyceraldehyde-3-phosphate dehydrogenase, which was found down-regulated (Table S1F), circumvents NADH- and ATP-producing steps of GL. The down-regulation shows that OA influences the redox-balance between plastids and the cytosol [52].

The down-regulation of callose synthase (GJ11230) and up-regulation of glucan beta-1,3-glucosidase (GJ14154) under OA indicates lowered buildup and enhanced degradation of chrysolaminaran-like glucans. OA also stimulated genes involved in catabolism of lipid compounds, e.g., the mitochondrial trifunctional enzyme (alpha subunit, GJ17682), a fatty acyl-CoA synthetase (GJ01107), but also a tentative polyketide synthase (GJ01666). In contrast to the diplont, mitochondrial beta-oxidation is induced indicating increased degradation of lipid storage compounds [53] under OA. While OA did not significantly affect cellular POC production on a phenomenological basis [16], which was interpreted as a physiological acclimation to maintain homeostasis, acquired transcriptomic data show that in particular the catabolic processes of carbon metabolism were strongly influenced by OA (Fig. 2A; Table S1F) and thereby counteracted stimulations in cellular POC production.

Regarding light reactions, a concerted down-regulation of genes related to chlorophyll synthesis, e.g., glutamate-1-semialdehyde aminomutase (GJ01010), porphobilinogen deaminase (GJ01909), uroporphyrinogen decarboxylases (GJ02406, GJ02644) and Mg-protoporphyrin O-methyltransferase (GJ14244) was observed in response to OA. Like in the diplont, this may be interpreted as a measure to decrease reductive pressure in photosynthetic light reactions. It is also in line with the lowered Chl *a*:POC ratios, i.e., the increased energy-efficiency observed under OA [16], and the hypothesis that cells need to reduce plastidic NADPH production to counteract the effects of OA-induced PPP activation. These findings show that also the haploid stage experiences a reconstellation of carbon fluxes between GL and PPP and consequently altered pool sizes and redox equilibria of NAD and NADP. The haploid cells, however, apply additional machinery to compensate for the alterations, namely by increasing catabolism of storage compounds, using unconventional fermentative pathways, and by lowering the rate of photosynthetic light and dark reactions. These seem to be the causes for the stability of POC production in the haplont under OA [16].

Genes involved in plasmalemmal signaling, e.g., PIPKs (GJ04870, GJ03851 and GJ00119), sphingosine-1-kinase (GJ24982) and associated downstream signaling kinases (GJ14428, GJ01704) were prominently up-regulated under OA (Fig. 2A; Table S1F). This indicates that, also in the haploid stage, OA affects environmental sensing and thus the control over cellular Ca^2+^ fluxes. Regarding ion fluxes, OA induced up-regulation of ion exchangers, e.g., a Ca^2+^/H^+^ antiporter VCX1 (GJ04544), a Na^+/^H^+^ exchanger (GJ13239) and a voltage-dependent Na^+^/Ca^2+^ channel VDC1 (GJ18307). The HCO_3_^−^ transporter with the Ca^2+^ binding motif (GJ14380) was found down-regulated in response to OA also in the haplont. Similar to the situation in the diplont, the differential expression of transporters must be triggered by the altered membrane gradients under OA. Following the interpretation that diminished calcification benefits biomass buildup in the diplont [16], it can be argued that the absence of calcification in the haplont, i.e., the absence of a process competing for C_i_, does not permit any stimulation of POC production under OA.

#### Energy-modulation of OA-responses

To examine the energy-modulation of OA-effects in the haplont, subset F was subtracted from subsets D and E, yielding only those OA-responses that occur exclusively under either low or high light levels. These subsets of genes responded to OA in an energy-dependent manner (i.e., *low-light-specific* and *high-light-specific* OA-responses; Fig. 1D and 1F, 1E and 1F; Fig. 2B, 2C), which likely underlies the observed light-dependent modulation of OA responses [16].

The low-light specific OA-response showed significant regulation of 491 genes (332↑ and 159↓; Fig. 1D and 1F; Table S1D and S1F). Visual inspection revealed 62 genes (46↑ and 16↓) that could be assigned to the categories of interest (Fig. 2B). Regarding carbon metabolism, three carbonic anhydrases (GJ19123, GJ04992, GJ1572) as well as cytoplasmic glyceraldehyde-3-phosphate dehydrogenase (GJ03426) and a lactate dehydrogenase (GJ11998) were up-regulated under OA when light intensity was low. A putative cytoplasmic fructose-2,6-bisphosphatase (GJ04538) and a plastidic glyceraldehyde-3-phosphate dehydrogenase (GJ26483) were found down-regulated. Such differential expression of key enzymes of the carbon metabolism suggests that haploid cells make use of unconventional pathways to control redox-balance (e.g., lactate fermentation, non-phosphorylating glyceraldehyde-3-phosphate dehydrogenases). Concerning light reactions, signaling and ion fluxes, no indicative regulation patterns could be interpreted in this low-light-specific OA-response.

In high-light acclimated haploid cells, OA caused additional expression of 1356 genes (628↑ and 728↓; Fig. 1E and 1F; Table S1E and S1F). Visual inspection revealed 111 significantly regulated genes (52↑ and 59↓) that could be assigned to the categories of interest (Fig. 2C). Regarding carbon metabolism, down-regulation of glycerol-3-phosphate dehydrogenase (GJ02661) was observed, indicating decreased activity of the glycerol-3-phosphate-shuttle system that conveys cytosolic NADH into the mitochondrial respiratory chain. Genes related to carboxylating enzymes were also OA-sensitive especially under high light, namely there was an up-regulation of two isoforms of phosphoenolpyruvate carboxykinase (GJ00405, GJ01561) and down-regulation of pyruvate carboxylase (GJ26507). These anaplerotically carbon-fixing enzymes are thought to primarily provide carbon skeletons for amino acids synthesis in the diploid stage [54]. Indeed, several genes of the amino acid metabolism were prominently regulated in the high-light-specific OA response (Table S1E and S1F). Interestingly, genes related to fatty acid degradation, e.g., hydroxyacyl-CoA dehydrogenase (GJ00215), long-chain acyl-CoA synthetase (GJ22967) and 3-oxoacyl-CoA thiolase (GJ01189) were up-regulated under OA. In addition, expression of the glyoxylate pathway enzymes malate synthase G (GJ00164) and isocitrate lyase (GJ00349) was significantly increased. This pathway provides the tricarboxylic acid cycle with carbon skeletons derived from fatty acid beta-oxidation, strongly suggesting an increased utilization of lipid compounds in the haploid stage that was indicated previously [55]. Also, two beta-1,3-glucan hydrolases (GJ13343, GJ13856) were found up-regulated, indicating an enhanced OA-induced degradation of chrysolaminaran-like glucans. Regarding light reactions, uroporphyrinogen decarboxylase (GJ03150) and violaxanthin de-epoxidase (GJ02541) were down-regulated. Besides the down-regulation of two vacuolar pyrophosphate-dependent H^+^-translocators (GJ03805, GJ18762), OA induced no further pronounced expression shifts in the categories ‘signaling’ or ‘ion fluxes’ under high light intensity.

Apparently, when haploid cells experience OA under scarce energy, tighter regulation of carbon fluxes and redox-balance is required. Under saturating light intensities, cells reduce the photosynthetic pressure of reduction equivalents and additionally intensify the breakdown of lipids and carbohydrates.

### Stage-specific Utilization of Genetic Inventory

In view of the stage-specific core OA-responses discussed above, it becomes obvious that there are the certain functional responses to OA in both stages (e.g. affection of the PPP, signaling, ion-homeostasis). However, only few functions appear in the subset that contains the *stage-independent* responses: The stage-specific core responses to OA (Fig. 1C, 1F) were overlapped across stages, yielding those genes that react uniformly in both life-cycle stages (Fig. 1G; Table S1G). The stage-independent OA-response included 207 genes (133↑ and 74↓). Visual inspection revealed a significant regulation of 26 genes (19↑ and 7↓) that could be sorted into the categories of interest (Fig. 2E). Regarding carbon metabolism, both stages up-regulated a putative glucan beta-1,3-glucosidase (GJ14154) and a generic beta-galactosidase (GJ01691). Up-regulation of the PPP-enzyme 6-phosphoglucono-lactonase (GJ20503) and a 3-oxoacyl-(ACP) synthase involved in fatty acid synthesis (GJ00191) was observed. Regarding light reactions, no common OA-response could be found in the stages. With respect to signal transduction, OA induced up-regulation of a PIPK (GJ04870), a sphingosine-1-kinase (GJ24982), and downstream kinases, i.e., CBL-interacting protein kinases (GJ01704, GJ14428). Concerning ion fluxes, there was common up-regulation of a hyperpolarization-activated, cyclic nucleotide-gated K^+^/Na^+^ channel (GJ12789) as well as down-regulation of a voltage-gated Ca^2+^ channel (GJ05176). The putative HCO_3_^−^ transporter with the Ca^2+^-binding EF-hand motif (GJ14380) was found down-regulated in response to OA in both stages.

Apparently, although both stages regulate ∼1200–1400 genes in response to OA, there are comparably few common genes used (∼200; Fig. 1G, Table S1G). This strongly advocates the presence of a tripartite genome in *E. huxleyi*, of which one general part is constitutively expressed, while two parts are selectively expressed depending on the ploidy stage [56]. This stage-specific utilization of the genome is even more obvious when regarding the common response to high light intensities (Fig. 1N). These ploidy-specific genes products are most likely those that determine the distinct functionalities of the life-cycle stages and enable their evolutionary success in the contemporary oceans.

### Treatment-specific Utilization of Genetic Inventory

It could be shown for both stages that the transcriptomic responses to OA (Fig. 1C, 1F) did not resemble the responses to high light intensity (Fig. 1J, 1M), as was previously hypothesized based on pigment contents and physiological data [16]. In fact, only a very limited number of genes was found uniformly regulated in response to both, OA and light (Fig. 1O, 1P; Fig. 2D; Table S1O, S1P), and those did not even reflect the functional responses discussed above. Apparently, OA and high light-intensities invoke regulation of *different* genes, which are, however, involved in the *same* pathways of the carbon metabolism (e.g., GL, PPP, lipid metabolism). Such mechanisms allow for the integration of qualitatively different environmental signals (e.g., [CO_2_], light) at the level of biochemical pathways. Thereby, the activity of the same physiological processes can be adjusted in response to multiple environmental variables. Such regulation schemes reflect the decentralized organization of metabolic networks, which is likely responsible for synergistic or compensatory effects of environmental stressors and may explain the similarity in phenomenological effects observed in response to high light and OA [16].

### Carbon Concentrating Mechanisms

It was previously concluded that C_i_ acquisition in the diplont was insensitive to OA because the preferred carbon source (∼80% HCO_3_^−^) and the high affinities for DIC were unaffected by the treatment [16]. Here, the observed regulation of uptake machinery in the diplont (i.e., 4 HCO_3_^–^transporters and 1 beta-carbonic anhydrase) shows, however, that an alteration of C_i_ acquisition indeed occurs, at least on the transcriptomic level. The apparent OA-insensitivity of HCO_3_^−^ usage and uptake affinity observed previously may derive from instantaneous pH effects, as these measurements are performed under stabilized pH conditions that often do not mimic environmental situations. In the haplont, the major carbon source was also not influenced (∼80% HCO_3_^−^) but uptake affinities were decreased, indicating altered CCM activity under OA [16]. Transcriptomic data show that a plastid-targeted HCO_3_^–^transporter is down-regulated under OA, together with energy-dependent regulation of four distinct carbonic anhydrases of the beta and delta-type (Table S1). Facing the large number of genes encoding HCO_3_^–^transporters and CAs (>12 and >7, respectively in CCMP1516; www.jgi.doe.gov) and their unknown cellular localization, it is challenging to derive conclusive statements about stage-specific CCM regulation in *E. huxleyi*. It can be concluded, however, that both stages apply distinct CCMs consisting of *different* modular components that complement the *same* plastidic Ca^2+^-sensitive HCO_3_^–^transporter. Such coordinated expression and localization of active and passive CCM components may indeed represent differently cost-intensive modes of C_i_ acquisition.

### Conclusion

In both stages, OA affected expression of genes involved in central carbon metabolism (GL, PPP, lipid and glucan turnover) as well as light physiology (light harvesting, xanthophyll cycling). This leads to altered fluxes of carbon and energy within and across compartments. The combined effects of OA and light on these fluxes originate from their feedback-interaction with the redox equilibria of NAD and NADP, which constitute a primal sensor- and control system in prokaryotic and eukaryotic cells [30]. In other words, OA affects cellular redox-state as a master regulator and thereby causes energy-dependent reconstellations of metabolic flux networks (Fig. 3). Another commonality of both life-cycle stages was the OA-sensitivity of signal-transduction mechanisms and ion fluxes, processes being major controllers of the cellular Ca^2+^ messenger system. Regulation of these processes may compensate altered signal-processing and offset membrane gradients under OA. Interestingly, no energy-modulation of these OA-responses (Fig. 2B, 2C; Table S1) could be observed. In line with this, the absence of related genes from the light core-responses of both stages (Fig. 1J, 1M) emphasizes that OA, but not light, affects cellular signaling and ion fluxes. Obviously, these processes are independent of energy supply, most likely because signaling cascades and ion fluxes need to be shielded from fluctuations in light intensity, as easily imposed by mixing and clouding. While the OA-responses of the stages are functionally similar, the haplont utilizes a genetic repertoire distinct from the diplont, which emphasizes the genetic and ecological flexibility of this organism. Still, the functional similarity of OA-responses suggests a general ‘mode of action’ of OA that may well occur in other protists. Future research should target effects of multiple stressors on the interaction of redox-balance with carbon metabolism as well as the relations between signaling and calcification.

## Materials and Methods

### Culture Conditions

Diploid and haploid *Emiliania huxleyi* (strains RCC 1216 and 1217, also known as TQ26 2N and 1N) were grown at 15°C in 0.2 *µ*m filtered North Sea seawater (salinity 32), enriched with vitamins and trace-metals (F/2 medium; [57]). Nitrate and phosphate were added in concentrations of 100 and 6.25 *µ*mol L^−1^, respectively. Cultures were grown under a 16∶8 h light:dark cycle with light intensities of 50 and 300 *µ*mol photons m^−2^s^−1^ (Biolux 965 daylight lamps, OSRAM, Germany), as measured with a datalogger (Li-Cor, Lincoln, USA) using a 4*π*-sensor (Walz, Effeltrich, Germany). Medium *p*CO_2_ was set by purging with humidified *p*CO_2_-adjusted gas mixtures (38.5 Pa and 101.3 Pa) for at least 16 h prior to inoculation. CO_2_-free air (<1 ppm CO_2_; Dominick Hunter, Willich, Germany) was mixed with pure CO_2_ (Air Liquide Deutschland, Düsseldorf, Germany) by a mass flow controller based system (CGM 2000 MCZ Umwelttechnik, Bad Nauheim, Germany). The *p*CO_2_ was regularly controlled with a non-dispersive infrared analyzer system (LI6252, Li-Cor) calibrated with CO_2_-free air and purchased air mixtures of 150±10 and 1000±20 ppm CO_2_ (Air Liquide, Düsseldorf, Germany). Prior to the experiment, cells were kept in exponential growth phase under experimental settings (light, *p*CO_2_) for at least two weeks in dilute batch cultures to assure proper acclimation. This was done to assess steady-state effects instead of transient shock responses. After inoculation, the 900 mL cylindrical flasks were continuously purged with the humidified *p*CO_2_-adjusted gas mixtures to avoid cell sedimentation and to minimize DIC depletion (flow-rate ∼130±10 mL min^−1^).

### Carbonate Chemistry

To ensure quasi-constant seawater carbonate chemistry (Table S2), only cultures in which the pH did not deviate more than 0.05 units from a cell-free medium were used for measurements (pH was measured with pH3000 microprocessor pH-meter; WTW, Weilheim, Germany; calibration was performed with NIST certified buffers). DIC was measured colorimetrically according to [58], using a TRAACS CS800 autoanalyzer (Seal Analytical, Norderstedt, Germany). Total alkalinity was calculated from linear Gran-titration plots [59], which were produced using an automated burette system (TitroLine alpha plus, Schott, Mainz, Germany). Calculations of carbonate chemistry were performed using CO_2_SYS [60] and were based on measurements of pH (NBS scale), total alkalinity, temperature and salinity. For the calculations, phosphate concentrations of 4 *µ*mol L^−1^ were assumed. The carbonic acid dissociation constants obtained by Mehrbach et al. ([61] refit by [62]) and those of sulfuric acid obtained by Dickson [63] were used.

### RNA Sampling

Acclimated cells were harvested between 4 and 8 h after the beginning of the light period at densities of 50.000–90.000 cells ml^−1^, as assessed with a Multisizer III hemocytometer (Beckman-Coulter, Fullerton, USA). For sampling, ∼1.5*10^7^ cells were concentrated by filtration (1.2 µm polycarbonate filters, Millipore, Billerica, USA) and pelleted by 5 minute centrifugation at 5000 g in a table centrifuge (Hettich, Bäch, Switzerland). Cell disruption was performed with a beadmill (Qiagen, Hilden, Germany) after adding 100 µL glassbeads (0.1 mm). Lysate was homogenized using QIAshredder spin-columns (Qiagen). RNA extraction was performed using a silica-column based guanidinium thiocyanate method (RNeasy mini, Qiagen). To digest DNA in the isolate, 7 Kunitz units of bovine DNase I (Qiagen) were applied to the silica matrix for 20 minutes at room temperature. After elution, MicroCon YM 30 ultrafiltration columns (Millipore) were used to further enrich RNA. Concentration and purity of the RNA were measured photometrically with a Nanodrop ND1000 (PeqLab, Erlangen, Germany) and integrity of the isolate was verified using a Bioanalyzer 2100 (Agilent, Waldbronn, Germany) running an RNA 6000 Nano LabChip (Agilent).

### Microarray Hybridizations

Microarrays were used as part of a standardized in-house working-pipeline. This assures reproducible sample processing and robust data analysis, not only between methods (e.g., in comparison with qRT-PCR; [55]) but also between experiments, which is not given with RNA-Sequencing approaches [64]. RNA Spike-In Mix (Agilent, p/n 5188–5279) was added to the RNA samples prior to the labeling reactions as an internal standard and benchmark for hybridization performance (Agilent RNA Spike-In Kit protocol). 200 ng total RNA from samples was reversely transcribed, and resulting cDNA was amplified as labeled cRNA (Two-color low RNA Input fluorescent linear amplification kit, Agilent, p/n 5184–3523). Incorporation of Cy-3 and Cy-5 labeled cytidine 5′-triphosphate (Perkin Elmer, Waltham, USA) into the cRNA and the control (pooled RNA from various treatments) was verified photometrically using the NanoDrop ND1000 (PeqLab). Labeling efficiencies were calculated as pmol dye (ng cRNA)^−1^ from the results of photometry and were in the range of 0.013 - 0.018 pmol dye (ng cRNA)^−1^. Microarray hybridizations were carried out in SureHyb hybridization chambers (Agilent, p/n G2534A). Every biological replicate was hybridized against a pooled, common control baseline to minimize hybridization biases. Treatment-vs.-treatment expression ratios were then calculated from the single treatment-vs.-control expression ratios. 750 ng of each Cy-3 and Cy-5 labeled cRNA was hybridized to 2×105K *Emiliania huxleyi* custom-built microarrays (Agilent). Three microarray probes were designed for each of 28670 transcript clusters (i.e. on-chip technical replication). The transcript clusters included sequences obtained by Sanger sequencing (Von Dassow et al. 2009) as well as ESTs compiled from the *E. huxleyi* CCMP1516 genome-project, conducted by the Joint Genome Institute (JGI; www.jgi.doe.gov). Probe design was done using Agilent’s eArray online platform. Following the Two-Color Microarray-based Gene Expression Analysis protocol (Agilent, p/n 5188–5242), hybridization was performed in a hybridization oven at 65°C for 17 hours at an agitation of 6 rpm. After hybridization, microarrays were disassembled in Wash Buffer 1 (Agilent, p/n 5188–5325), washed with Wash Buffer 1, Wash Buffer 2 (Agilent, p/n 5188–5326), acetonitrile (VWR, Darmstadt, Germany) and ‘Stabilization and Drying Solution‘ (Agilent, p/n 5185–5979) according to manufacturer’s instructions. Stabilization and Drying Solution, an ozone scavenger, protects the Cy-dye signal from degradation. Arrays were immediately scanned with a G2505C microarray scanner (Agilent) using standard photomultiplier tube settings and 5 µm scan resolution.

### Data Generation

Raw data was extracted with Feature Extraction Software version 9.0 (Agilent), incorporating the GE2_105_Dec08 protocol. Array quality was monitored using the QC Tool v1.0 (Agilent) with the metric set GE2_QCMT_Feb07. Analysis was performed using GeneSpring 11 (Agilent). LOWESS-normalized data were submitted to the MIAMExpress database hosted by the European Bioinformatics Institute (EBI; www.ebi.ac.uk/arrayexpress; accession code E-MEXP-3624). Hybridization results of biological triplicates (i.e., treatment-vs.-treatment expression ratios) were evaluated in multiple comparison tests using ANOVA. Regulation was called significant when probe-specific *p*-values were ≤0.05. The dataset was then reduced to only those genes in which expression changed ≥1.5 fold in response to the treatment. When a divergent regulation was reported, i.e., one or more probes for the same transcript cluster indicating regulation in opposite directions, the respective probe set was as a whole excluded from further analyses (<12 probe sets per hybridization). In case that only one out of three probes reported significant differential expression but two probes reported unaltered expression, the respective probe sets were as well excluded from further analyses (300–700 probe sets per hybridization) to increase the confidence level of results. The remaining probe sets were merged and reported as one significantly regulated transcript cluster. For completeness, the number of hit probes and the averaged fold-change is reported for the transcript clusters (Table S1).

### Gene Annotation

Significantly regulated transcripts were assigned to an annotation table. This table was generated by using BLASTn similarity searches, in which the ∼28670 transcript clusters were aligned with the ‘Emihu1_best_transcripts’ database provided by the JGI. After excluding alignments with an e-value >10^−5^, the two best aligning, but different transcript models were implemented into the annotation table. This allowed the assignment of ∼21740 investigated transcript clusters to models existing in the JGI *E. huxleyi* gene catalog. Assigned JGI protein IDs were then aligned with ‘best gene-model’ predictions, based on similarity to eukaryotic orthologous genes (KOG; provided by the JGI). This KOG-database harbors functional information on ∼11930 different *E. huxleyi* models in the JGI catalog. Additionally, generic gene information obtained by Blast2GO (B2G) queries of all clusters ([65]; e-value cutoff at >10^−6^) was augmented to the annotation table. The final transcriptome screening involved ∼10000 unique *E. huxleyi* gene models with a confidently predicted function.

### Dataset Evaluation

Acquired datasets with significantly regulated clusters and their associated annotations were partitioned with Venn diagrams to compare the gene expression patterns occurring in response to the treatments. Subsets were manually inspected towards expression patterns that give information on metabolic pathways or processes being regulated in response to the applied treatments. To overcome the problematic gap between crude gene expression patterns and enzyme activities [66], [67] and to increase confidence in results, all findings were discussed in accordance with phenomenological and physiological observations obtained with independent methodologies [16]. Genes of interest were sorted into the *sensu*-*latu* categories ‘carbon metabolism’ (including turnover of hydrocarbons and carbohydrates), ‘light reactions’ (including pigment turnover), ‘signaling’, as well as ‘inorganic ion transport’. After categorization, integrity and validity of the gene models of interest were reconfirmed by model inspection in the JGI draft genome database and by BLAST searches. In the text, exemplary transcripts are notated with their numerical cluster identifiers. JGI identifiers of the associated protein and complete expression datasets can be taken from supplementary spreadsheet file (Table S1).

## Supporting Information

Table S1 **Gene expression data on the effects of Ocean Acidifcation in diploid and haploid** ***Emiliania huxleyi*** **(RCC 1216/1217) under limiting and saturating light intensities.** For completeness, the spreadsheet holds information on the quality of obtained BLAST and Blast2GO alignments.
(XLSX)

Table S2 **Attained seawater chemistry during cell culture.** CO_2_ partial pressure (pCO_2_), concentrations of dissolved inorganic carbon, bicarbonate and carbonate (DIC, HCO_3_^−^, CO_3_^2−^) and calcite saturation state (Ω_calcite_) were calculated based on pH_NBS_ and total alkalinity (TA) using CO_2_SYS [60]. ‘Reference’ denotes carbonate chemistry of cell-free seawater. Results are reported for 15°C (n ≥3; ± SD).
(DOCX)
