# Supplementary material for: Ocean Acidification Affects Redox-Balance and Ion-Homeostasis in the Life-Cycle Stages of Emiliania huxleyi
Source: PLoS One. 2012 Dec 26;7(12):e52212. doi: 10.1371/journal.pone.0052212 (PMC3530605; doi:10.1371/journal.pone.0052212)
Supplement: Table S2 — Attained seawater chemistry during cell culture. CO2 partial pressure (pCO2), concentrations of dissolved inorganic carbon, bicarbonate and carbonate (DIC, HCO3 −, CO3 2−) and calcite saturation state (Ωcalcite) were calculated based on pHNBS and total alkalinity (TA) using CO2SYS [60]. ‘Reference’ denotes carbonate chemistry of cell-free seawater. Results are reported for 15°C (n ≥3; ± SD). (DOCX) [file pone.0052212.s002.docx]

**Supplementary table S2**

| Strain, ploidy | Photon flux  (µmol m^-2^ s^-1^) | Treatment *p*CO_2_ (Pa) | Attained  *p*CO_2_ (Pa) | pH_NBS_ | TA  (µmol kg^-1^) | DIC  (µmol kg^-1^) | HCO_3_^-^  (µmol kg^-1^) | CO_3_^2-^  (µmol kg^-1^) | Ω_calcite_ |
| --- | --- | --- | --- | --- | --- | --- | --- | --- | --- |
| RCC 1216,  2N | 50 | low, 38.5 | 40.4 ± 0.4 | 8.112 ± 0.014 | 2254 ± 20 | 1997 ± 44 | 1910 ± 17 | 140.1 ± 2.1 | 3.47 ± 0.05 |
|  |  | high, 101.3 | 97.3 ± 11.1 | 7.808 ± 0.012 | 2385 ± 7 | 2232 ± 22 | 2186 ± 1 | 80.0 ± 2.8 | 1.94 ± 0.07 |
|  | 300 | low, 38.5 | 43.5 ± 0.2 | 8.083 ± 0.040 | 2273 ± 5 | 2018 ± 15 | 1942 ± 4 | 134.6 ± 0.6 | 3.33 ± 0.01 |
|  |  | high, 101.3 | 110.6 ± 14.6 | 7.741 ± 0.002 | 2322 ± 35 | 2174 ± 32 | 2149 ± 36 | 68.8 ± 1.9 | 1.67 ± 0.05 |
| RCC 1217,  1N | 50 | low, 38.5 | 35.6 ± 0.4 | 8.176 ± 0.005 | 2393 ± 24 | 2111 ± 19 | 1978 ± 21 | 170.8 ± 2.8 | 4.23 ± 0.07 |
|  |  | high, 101.3 | 95.7 ± 1.9 | 7.823 ± 0.022 | 2398 ± 3 | 2249 ± 23 | 2190 ± 4 | 83.5 ± 1.5 | 2.03 ± 0.04 |
|  | 300 | low, 38.5 | 35.5 ± 0.4 | 8.177 ± 0.019 | 2380 ± 10 | 2096 ± 24 | 1967 ± 18 | 169.5 ± 3.4 | 4.20 ± 0.08 |
|  |  | high, 101.3 | 95.2 ± 5.9 | 7.818 ±0.038 | 2402 ± 21 | 2296 ± 34 | 2199 ± 14 | 81.7 ± 7.1 | 1.98 ± 0.17 |
| Reference | - | low, 38.5 | 40.9 ± 1.2 | 8.134 ± 0.010 | 2411 ± 13 | 2134 ± 25 | 2030 ± 3 | 156.7 ± 4.9 | 3.88 ± 0.12 |
|  |  | high, 101.3 | 100.9 ± 10.4 | 7.770 ± 0.025 | 2396 ± 17 | 2269 ± 45 | 2171 ± 4 | 78.2 ± 7.7 | 1.90 ± 0.19 |
